# Biological and Chemical Processes of Nitrate Reduction and Ferrous Oxidation Mediated by *Shewanella oneidensis* MR-1

**DOI:** 10.3390/microorganisms12122454

**Published:** 2024-11-29

**Authors:** Lingyu Hou, Xiangyu Bai, Zihe Sima, Jiani Zhang, Luyao Yan, Ding Li, Yongguang Jiang

**Affiliations:** 1 School of Environmental Studies, China University of Geosciences (Wuhan), Wuhan 430078, China; hurly@cug.edu.cn (L.H.);; 2 Wuhan Institute of Biological Products Co., Ltd., Wuhan 430207, China; baixy1010@163.com (X.B.);; 3 College of Ocean and Earth Sciences, Xiamen University, Xiamen 361102, China

**Keywords:** *Shewanella oneidensis* MR-1, divalent iron, nitrate reduction, ferrous oxidation, biological and chemical processes

## Abstract

Iron, Earth’s most abundant redox-active metal, undergoes both abiotic and microbial redox reactions that regulate the formation, transformation, and dissolution of iron minerals. The electron transfer between ferrous iron (Fe(II)) and ferric iron (Fe(III)) is critical for mineral dynamics, pollutant remediation, and global biogeochemical cycling. Bacteria play a significant role, especially in anaerobic Fe(II) oxidation, contributing to Fe(III) mineral formation in oxygen-depleted environments. In iron-rich, neutral anaerobic settings, microbial nitrate-reducing Fe(II) oxidation (NRFO) and iron reduction processes happen simultaneously. This study used *Shewanella oneidensis* MR-1 to create an anaerobic NRFO system between Fe(II) and nitrate, revealing concurrent Fe(II) oxidation and nitrate reduction. Both gene-mediated biological Fe(II) oxidation and chemical Fe(II) oxidation, facilitated by nitrite (a byproduct of nitrate reduction), were observed. The *MtrABC* gene cluster was linked to this process. At low Fe(II) concentrations, toxicity and mineral precipitation inhibited nitrate reduction by *Shewanella oneidensis* MR-1, whereas high Fe(II) levels led to Fe(II) oxidation, resulting in cell encrustation, which further constrained nitrate reduction.

## 1. Introduction

Many habitats on Earth are not rich in oxygen. For instance, numerous oceanic and lacustrine regions exhibit seasonal or permanent hypoxia. In soils and sediments, oxygen typically penetrates only a few millimeters, particularly in waterlogged, organic-rich environments [1]. Similarly, deep-sea and continental crustal areas are typically oxygen-deficient. The discovery of anaerobic Fe(II)-oxidizing microorganisms expands our understanding of Fe(II) oxidation in previously underexplored environments, highlighting the ecological significance of these bacteria [2]. Several strains of nitrate-reducing Fe(II)-oxidizing microorganisms (NRFeOx) and phototrophic Fe(II)-oxidizing microorganisms (pFeOx) have been isolated, and genomic, metagenomic, and metatranscriptomic studies offer further insights [3,4]. However, compared to our understanding of microbial Fe(III) reduction and microaerophilic Fe(II) oxidation, the ecological roles, physiological traits, and enzymatic mechanisms of anaerobic Fe(II) oxidation remain largely unexplored [5]. These microorganisms possess unique physiological adaptations in low-oxygen environments, which differ significantly from those in oxic conditions, resulting in distinct interactions with their surroundings [6]. To fully understand their impact on Earth’s systems, it is crucial to investigate iron cycling processes under anoxic conditions [7].

The coupled reduction of nitrate and Fe(II) oxidation was first observed over 20 years ago, leading to the recognition that nitrate-reducing bacteria interact with Fe(II) through distinct mechanisms [8]. First, autotrophic nitrate-reducing Fe(II)-oxidizing bacteria (NRFeOx) utilize Fe(II) as an electron donor to generate energy and fix carbon dioxide, thereby synthesizing biomass without requiring organic carbon [9]. In contrast, most nitrate-reducing bacteria require organic substrates, such as acetate, to sustain Fe(II) oxidation and are termed “mixotrophic” microorganisms, utilizing both organic carbon and Fe(II) as electron donors [10]. Additionally, some Fe(II) oxidation is thought to result from abiotic reactions between Fe(II) and intermediates of heterotrophic denitrification, such as nitrite or nitric oxide [11]. In this study, organisms that oxidize Fe(II) while reducing nitrate without enzymatic evidence are classified as “chemodenitrifiers”, rather than true nitrate-reducing Fe(II)-oxidizers. This reaction pathway is frequently observed in classic denitrifiers, such as Escherichia coli, and it is expected that this classification applies broadly to heterotrophic denitrifiers that grow in high Fe(II) concentrations [12]. Notably, many autotrophic NRFeOx strains struggle to sustain Fe(II) oxidation without supplementary carbon sources. Furthermore, in strains requiring organic carbon, whether Fe(II) oxidation involves enzymatic processes—classifying them as “mixotrophic” or “chemodenitrifiers”—remains unclear [13].

The mechanisms of Fe(II) oxidation by phototrophic Fe(II)-oxidizing bacteria under anoxic conditions are not fully understood, particularly regarding the oxidation of dissolved Fe(II), ligand-bound Fe(II), and solid-phase minerals [14]. Furthermore, the mechanisms by which these bacteria process iron from solid mineral precipitates remain unclear. Research on the phototrophic Fe(II) oxidation pathway has primarily focused on *Rhodopseudomonas palustris* TIE-1 [15]. In this bacterium, electron transfer is mediated by the pioABC operon, which encodes a periplasmic decaheme c-type cytochrome (PioA), an outer membrane porin-like protein (PioB), and a periplasmic iron–sulfur cluster protein (PioC) [16]. PioA and PioB are homologous to MtrA and MtrB proteins in *Shewanella oneidensis* MR-1, a well-known Fe(III)-reducing bacterium [15]. Deletion of PioA in TIE-1 nearly completely inhibits Fe(II) oxidation, whereas deletions of PioB and PioC only partially reduce this capacity [16]. Additionally, TIE-1 selectively oxidizes surface-bound Fe(II) on magnetite, suggesting that its oxidation mechanism may involve direct cell–mineral contact [17].

Anaerobic Fe(II)-oxidizing bacteria are both environmentally significant and promising for biotechnological applications. For instance, Fe(II)-oxidizing bacteria can enhance nitrate removal in wastewater treatment facilities, improving potable water, wastewater, and sludge management [18]. This process may occur naturally in aquifers, where anaerobic Fe(II)-oxidizing bacteria facilitate nitrate reduction by oxidizing Fe(II)-rich clays or minerals, such as pyrite [19]. Moreover, minerals produced by anaerobic Fe(II)-oxidizing bacteria, particularly active Fe(II)-Fe(III) species like green rust or magnetite, may contribute to metal and pollutant remediation [20]. The role of biogenic iron minerals in controlling arsenic (As) mobility is well documented [21]. In anoxic Fe- and As-rich systems, such as rice paddy soils or As-contaminated aquifers, iron mineral formation sequesters arsenic and limits its dispersal. Although the significance of biogenic Fe(III) minerals in As-contaminated aquifers is not fully understood, the adsorption properties of iron are utilized in drinking water filters in As-rich areas, offering a cost-effective filtration solution [21]. On one hand, Fe(II)-oxidizing bacteria may aid in arsenic removal through co-precipitation; on the other hand, studies suggest that arsenic binds less effectively to biogenic Fe(III) minerals compared to abiotic Fe(III) minerals, possibly due to competition from bacterial organic matter for surface adsorption [22].

Nitrite (NO_2_^−^) is a significant source of surface and groundwater pollution, contributing to eutrophication, promoting excessive algal growth, depleting dissolved oxygen in water, and ultimately endangering aquatic ecosystems [23]. Additionally, nitrite can reduce the oxygen-carrying capacity of blood, posing particular risks to infants and fish, and may form carcinogenic compounds in the body [24]. Research by Alfei et al. has demonstrated that cationic polystyrene-based hydrogels can efficiently remove nitrites from water through electrostatic interactions [25]. The widespread presence of nitrite as a water pollutant highlights not only the inadequacies in agricultural and domestic wastewater management but also the critical importance of developing efficient removal technologies. Effectively controlling nitrite pollution is essential for protecting aquatic ecosystems and public health.

Extensive research on the two types of anaerobic Fe(II)-oxidizing bacteria has yielded foundational insights into their physiology, Fe(II) oxidation mechanisms, and environmental roles. However, further studies are needed to deepen our understanding. From a physiological perspective, it is essential to test proposed models of anaerobic Fe(II) oxidation mechanisms to assess their prevalence and variability. Moreover, further research is required to evaluate how growth conditions influence the mineral characteristics formed by Fe(II) oxidizers, particularly regarding why only certain isolates can oxidize solid substrates [26]. Few studies have investigated these bacteria under environmentally relevant conditions, such as in low iron concentrations or in the presence of competing substrates (organic or inorganic). However, such insights are crucial for translating laboratory findings into practical environmental applications.

## 2. Materials and Methods

### 2.1. Materials

The study specimen used in this experiment is *Shewanella oneidensis* MR-1 (ATCC^®^BAA-1096™), purchased from the United States model culture collection repository. The culture medium required for the culture of *Shewanella oneidensis* is LB medium [L-1 tryptone, 10 g/L; L-1 yeast extract, 5 g/L; L-1 sodium chloride, 10 g/L; pH = 7.0] [27,28]. Under test conditions, MR-1 was cultured on the M1 medium [10 mL of Vitamin Mix (biotin, 2 mg/L; folic acid, 2 mg/L; pyridoxine HCl, 10 mg/L; riboflavin, 5 mg/L; thiamine, 5 mg/L; nicotinic acid, 5 mg/L; pantothenic acid, 5 mg/L; B-12, 0.1 mg/L; p-aminobenzoic acid, 5 mg/L; thioctic acid, 5 mg/L); 10 mL of Amino Acid Mix (L-glutamic acid, 2 g/L; L-arginine, 2 g/L; D-L-Serine, 2 g/L); 10 mL of Mineral Mix (NTA, 1.5 g/L; MgSO_4_, 3 g/L; MnSO_4_·H_2_O, 0.5 g/L; NaCl, 1 g/L; FeSO_4_·7H_2_O, 0.1 g/L; CaCl_2_·2H_2_O, 0.1 g/L; CoCl_2_·6H_2_O, 0.1 g/L; ZnCl_2_, 0.13 g/L; CuSO_4_·5H_2_O, 0.01 g/L; AlK(SO_4_)_2_·12H_2_O, 0.01 g/L; H_3_BO_3_, 0.01 g/L; Na_2_MoO_4_, 0.025 g/L; NiCl_2_·6H_2_O, 0.025 g/L; Na_2_WO_4_·2H_2_O, 0.025 g/L); PIPES buffer (0.91 g/L; NaOH,0.3 g/L; NH_4_Cl, 1.5 g/L; KCl, 0.1 g/L; NaH_2_PO_4_·H_2_O, 0.6 g/L; Na_2_SO_4_, 0.213 g/L)] for anaerobic culture, in which the anaerobic gas is high-purity nitrogen (99.999%) [29,30]. The anaerobic bottle containing pure water (100mL) and the empty anaerobic bottle with the same capacity were connected to the deaeration device with 100% N_2_ for 30 min [31]. The anaerobic bottle was autoclaved at high temperature and high pressure. Subsequently, ferrous citrate (C_6_H_8_FeO_7_ 1 mol/L) was prepared in an anaerobic workstation and further sealed with an aluminum cover after being pressed with a blue butyl rubber stopper. The prepared ferrous citrate was balanced in an anaerobic workstation for 24 h and then filtered with a 0.22 μm filter head into an empty anaerobic bottle deoxygenated by high-temperature sterilization and stored in the dark. After that, fumaric acid (1 mol/L), lactate (1 mol/L), nitric acid (1 mol/L), nitrous acid (1 mol/L), and acetic acid (1 mol/L) were added.

### 2.2. Methods

The mutant strains in this study were constructed using the in-frame deletion method. Firstly, genomic DNA was extracted using a reagent kit, and two pairs of primers (5-O/3-I, 5-I/3-O) were designed. The upstream and downstream sequences of the target gene were amplified using Es taq polymerase and then fused into a long fragment using 5O/3O fusion PCR. After purification and enzymatic digestion, the product was inserted into the suicide plasmid pDS3.2 and then transformed into *E. coli* WM3064. Monoclonal antibodies identified as positive by PCR for subsequent conjugation transfer experiments were selected, and the final knockout strain through sucrose screening was obtained. Mutants Δ*MtrABC*, Δ*MtrDEF*, and Δ*MtrABCEDF* were obtained by individually knocking out the gene clusters *MtrABC* and *MtrDEF* of *Shewanella oneidensis* MR-1 cultured in an LB medium, as well as simultaneously knocking out *MtrABCDEF*. Similarly, mutants ΔdmsEFAB and *Δso4357–4360* were generated by knocking out *dmsEFAB* and *so4357–4360*. To put it simply, we employed polymerase chain reaction (PCR) to generate a DNA fragment encompassing only the 5’ and 3’ regions of the target gene cluster and subsequently cloned this DNA fragment onto the vector pDS3.2. Following this, the recombinant plasmid was transformed into Escherichia coli strain WM3064. Upon sequencing confirmation, the WM3064 strain harboring the recombinant plasmid was co-cultured with *Shewanella oneidensis* MR-1 for conjugative transfer. The recombinant fragment was then transferred into *Shewanella oneidensis* MR-1 via homologous recombination substitution. On LB agar plates containing GM, a single integron was selected, validated via PCR, and incubated overnight in an LB NaCl medium. Subsequently, sucrose was used for reverse selection of the deletion mutant on LB agar plates, which underwent double crossover to eliminate the pDS3.2 sequence [32]. Ultimately, the obtained mutants were verified through PCR and sequencing.

After overnight cultivation of MR-1 in liquid LB, transfer it to 50 mL of fresh liquid LB and continue culturing under the same conditions until the optical density (OD) reaches 1.2. Transfer 30 mL of the bacterial culture into a 50 mL centrifuge tube, centrifuge at 5000× *g* for 5 min at 25 °C, and discard the supernatant. Then, add 15 mL of sterile M1 medium, without electron donors or acceptors, to resuspend the bacterial cells. Conduct the aerobic growth experiments in 30 mL culture tubes, each containing a total volume of 15 mL. For anaerobic growth experiments, transfer the resuspended bacterial culture into a 30 mL anaerobic vial. Assemble the sterile deoxygenation needle and stopper on a clean bench and attach the sterilized 0.22 μm filter head to the inlet end of the deoxygenation needle. After assembly, connect it to the deoxygenation device and purge it with nitrogen for 10 min to remove oxygen [31]. Subsequently, add the resuspended bacterial solution to the prepared anaerobic M1 medium. Prior to inoculation, add various components to the M1 medium to achieve the desired working concentrations, as detailed in Section 1 and Section 2, including anaerobic ferrous citrate stock solution (20 mmol/L), sodium lactate (20 mmol/L), fumaric acid (20 mmol/L), acetic acid (15 mmol/L), citric acid (5 mmol/L), and nitric acid (10 mmol/L). Conduct the experiment in a 100 mL anaerobic flask containing 50 mL of total culture volume.

### 2.3. Experimental Conditions

#### 2.3.1. Selection of Experimental Strains

Anaerobic respiration is the primary mechanism by which bacteria participate directly in biogeochemical cycles. Bacteria can establish anaerobic respiratory electron transfer chains using various terminal electron acceptors, including inorganic compounds like nitrate and thiosulfate, organic compounds such as fumaric acid and dimethyl sulfoxide (DMSO), and metals like Fe(III) and Mn(IV) [32,33,34]. *Shewanella*, a Gram-negative bacterium in the class γ-*Proteobacteria*, is ubiquitous in water and sediment [34,35,36,37]. It can reduce a wide range of electron acceptors, including iron oxides, nitrates, and dimethyl sulfoxide (DMSO) [38]. Furthermore, as a model microorganism, *Shewanella oneidensis* MR-1 serves as an ideal subject for research [39,40].

**Figure 1:**
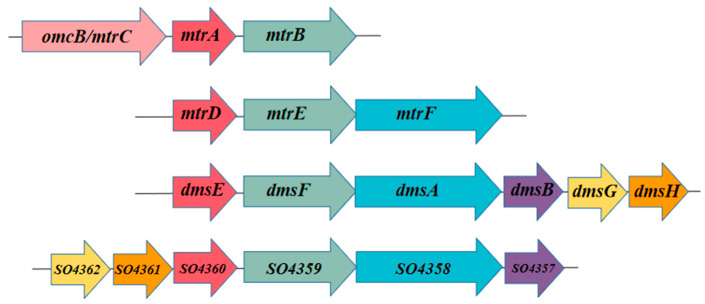
Extracellular electron transfer gene cluster of *Shewanella oneidensis* MR-1. The same color represents homologous genes. Two types of red, cytochrome protein genes; green, outer membrane porin gene; blue, containing molybdenum cofactor protein gene; purple, containing iron–sulfur central protein gene; and yellow and orange, companion protein genes.

To date, the metal reduction (Mtr) pathway of MR-1 is the most thoroughly understood extracellular electron transfer pathway [41]. The MR-1 genome contains four gene clusters encoding extracellular electron transfer channels (Figure 1) [34,42,43]. Specifically, *MtrABC* and *dmsEFAB* are involved in the reduction of metal oxides and DMSO, respectively [44,45]. Although the physiological roles of *MtrDEF* and the so4357-so4362 gene cluster remain unconfirmed, their sequences are homologies to *MtrABC* and *dmsEFAB*, suggesting similar functions at the molecular level.

In underground water pipeline networks susceptible to rust corrosion, various iron oxidation states coexist with compounds such as nitric acid and nitrite [46]. Due to their relatively high standard redox potentials, nitrate and nitrite serve as preferred electron acceptors under experimental conditions, with nitrate being favored [47]. Fumaric acid, a commonly used electron acceptor, is relatively stable and easy to control in anaerobic microbial metabolism research. Its moderate electron-accepting capacity allows it to participate in redox reactions with various electron donors, such as Fe(II), facilitating the observation of the Fe(II) oxidation in experimental setups. Under experimental conditions, fumaric acid is relatively resistant to side reactions, ensuring the accuracy of experimental results [48]. The use of fumaric acid helps establish a stable reducing environment in experiments, simulating biogeochemical processes under anaerobic conditions and aligning more closely with natural reaction mechanisms.

Studying the coupling of Fe(II) oxidation and nitrate reduction is of significant biological importance in groundwater and surface water environments. The redox cycle of Fe(II) and Fe(III) affects infrastructure stability [47]. During oxidation, Fe(II) is converted to Fe(III), which tends to undergo sedimentation in aquifers or underground pipelines, forming Fe(III) deposits [49]. These deposits accumulate on the surface of iron pipes in the groundwater pipeline system, causing corrosion and weakening structural integrity [50]. Consequently, this accelerates pipe aging and damage, increasing maintenance and replacement costs.

#### 2.3.2. Experimental Group Design

Based on the above analysis, fumaric acid (organic electron acceptor) and nitric acid (inorganic electron acceptor) were selected to establish the anaerobic Fe(II) oxidation electron transfer chain in MR-1. Initially, a series of tests were conducted on the MR-1 wild-type strain under conditions outlined in Table S1.

Initially, we examined the oxidation capacity of wild-type strains towards Fe(II) under specific conditions. Building on this, we constructed Δ*MtrABCDEF*, Δ*MtrDEF*, Δso4357–so4360, Δ*MtrABC*, and Δ*dmsEFAB* mutant strains as part of the experimental design outlined in Table S2. The second part of the experiment focused on the genetic control mechanism of the Fe(II) oxidation process. Using systematic gene knockout experiments, we focused on studying the *MtrABCDEF* gene cluster, the *dmsEFAB* gene, and the so4357–so4360 gene. These genes are believed to be closely related to Fe redox processes. The *MtrABCDEF* gene cluster has been reported as an important regulatory factor for iron reduction [51,52,53], while *dmsEFAB* and *so4357–so4360* were included due to their homology with *MtrABCDEF* [42,43]. By analyzing the Fe(II) oxidation capacity of these gene knockout strains, we aim to determine their specific roles and potential regulatory mechanisms in Fe(II) redox reactions.

### 2.4. Test Methods

#### 2.4.1. Determination of Ferrous Content

Take a 1.5 mL centrifuge tube and add 200 μL of 1M hydrochloric acid first, followed by 200 μL of the sample filtered through a 0.22 μm filter. Perform 1:1 acidification (to prevent Fe^2+^ oxidation), then vortex thoroughly. This step is equivalent to a twofold dilution. Subsequently, dilute the acidified sample with 0.5 M HCl, vortexing thoroughly after each dilution. Finally, add 900 μL of phenanthroline to 100 μL of the diluted sample, vortex thoroughly, and put 200 μL into the microplate reader well. Set the reader to 562 nm to measure absorbance. Plot the obtained absorbance value on the standard curve and multiply it by the dilution factor to determine the Fe(II) concentration [54,55].

#### 2.4.2. Determination of Nitric Acid and Nitrous Acid

The carbonate leaching solution system was selected for the determination of the sample extraction solution [56]. The concentrations of Na_2_CO_3_ and NaHCO_3_ in the leaching solution were 4.5 mmol/L and 0.8 mmol/L, respectively. The chromatographic conditions for sample detection are as follows: an IonPac AS23 anion analysis column (4 mm × 250 mm) paired with an IonPac AG23 protection column (4 mm × 50 mm) at a column temperature of 30 °C; an AERS-500 (4 mm) suppressor, capable of suppressing a current of 25 mA; a Dionex conductivity detector, with a detecting pool temperature of 35 °C; an isocratic elution using the leaching solution, with a flow rate of 1.0 mL/min. The injection volume is 25 μL, and the content of nitric acid and nitrous acid in the sample is calculated based on the peak areas of nitric acid and nitrous acid and the standard curve [56].

#### 2.4.3. Organic Acid Content Test

Shimadzu SPD-M20A high-performance liquid chromatography and an inert sustain C18 (5 μm, 4.6 × 250 nm) chromatography column were used to detect the changes in the content of organic acids such as cyclosuccinic acid, fumaric acid, and acetic acid [57]. The mobile phase was 0.1% phosphoric acid aqueous solution/methanol = 88:12 (V:V), the flow rate of the mobile phase was 0.5 mL·min^−1^, the column temperature was 30 °C, and the detector analysis wavelength was 210 nm. The sample was filtered through a 0.22 μm filter membrane into the inner liner tube of the injection vial, and the injection volume was set to 20 μL. After starting the program, the contents of succinic acid, fumaric acid, and acetic acid in the sample were calculated based on the peak areas of different organic acids and the standard curve.

## 3. Results

### 3.1. Shewanella oneidensis MR-1 WT Section

#### 3.1.1. MR-1 Ferrous Oxidation Curve Under Different Conditions

**Figure 2:**
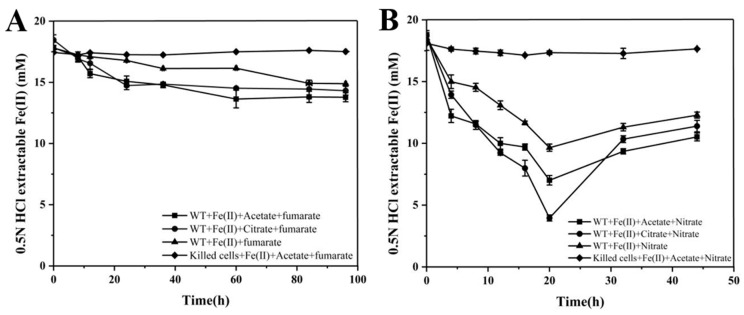
Ferrous oxidation curves of *Shewanella oneidensis* MR-1 under different conditions using fumaric acid (**A**) and nitric acid (**B**) as electron acceptors.

The results of WT + Fe(II) + acetate + fumarate (black square line) and WT + Fe(II) + citrate + fumarate (black circle) indicate that adding organic acids (acetic and citric acid) promotes iron oxidation. WT + Fe(II) + fumarate (black triangle) shows a slower rate of Fe(II) oxidation in the presence of fumarate alone. Killed cells + Fe(II) + acetate + fumarate (black diamonds) as the control group shows that dead cells contribute negligibly to Fe(II) oxidation, demonstrating that biological activity is crucial for Fe(II) oxidation. Overall, Fe(II) acts as an electron donor to reduce fumaric acid, which serves as an electron acceptor, while acetic acid, as a carbon source, significantly promotes iron oxidation. Whether citric acid acts as a carbon source or contributes as part of the electron donor requires further verification (Figure 2A). The effects of nitrite as an electron acceptor are illustrated by WT + Fe(II) + acetate + nitrite (black squares) and WT + Fe(II) + nitrite (black triangles), indicating the influence of carbon sources on iron oxidation in the presence of nitrite. WT + Fe(II) + citrate + nitrite (black circle) suggests that citric acid has a stronger promoting effect on Fe(II) oxidation than acetic acid. Killed cells + Fe(II) + acetate + nitrite (black diamonds) as a control showed almost no activity in iron oxidation, again emphasizing the importance of biological processes. At the same time, the change in trivalent iron content in the reaction system is calculated by the difference between the detected total iron content and divalent iron content, and the total iron content remains at the same level throughout the entire process. Therefore, the change in trivalent iron content is inversely proportional to the decrease in divalent iron content. Although iron oxidation begins rapidly under all conditions, the rate of iron concentration declines slowly over time. Around 20 h, Fe(III) in the system is reduced to Fe(II), directly related to the bacterial mediating effect (Figure 2B).

These two sets of experimental results highlight the roles of fumaric acid and nitric acid as electron acceptors in the MR-1 Fe(II) oxidation process, as well as the importance of biological activity. These findings provide valuable insights into the role of microorganisms in the iron cycling process. Further research could explore the specific effects of different concentrations of organic acids and nitric acid on Fe(II) oxidation, as well as how these conditions influence the metabolic activity of MR-1.

#### 3.1.2. The Effect of Nitric Acid on the Oxidation of Fe (II) and the Variation Curve of Nitric Acid

**Figure 3:**
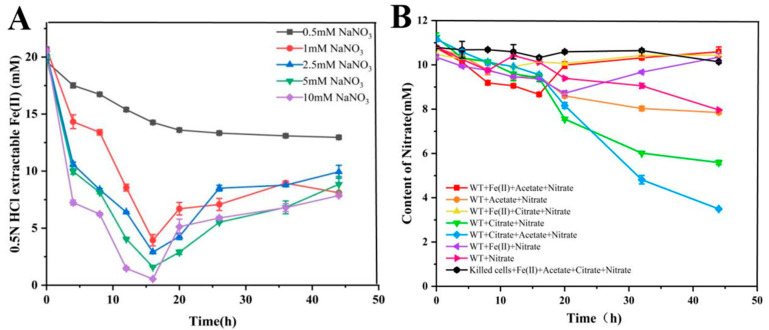
Determination of the optimal nitrate concentration of *Shewanella oneidensis* MR-1. The oxidation of ferrous ions at different concentrations of nitric acid (**A**) and the dynamic changes in the reduction in *Shewanella oneidensis* MR-1 at a nitrate concentration of 10 mM (**B**).

The effects of different concentrations of nitric acid on the oxidation of Fe(II) are as follows: At 0.5 mM NaNO_3_ (black line), the oxidation rate of Fe(II) is slower at lower concentrations. As the nitric acid concentration increases (1 mM, 2.5 mM, 5 mM, and 10 mM NaNO_3_, represented by red, blue, green, and purple lines, respectively), the Fe(II) oxidation rate accelerates, with a more rapid decrease in iron concentration. The iron concentration decreased most significantly at higher concentrations (5 mM and 10 mM NaNO_3_) after 20 h but stabilized after 30 h, indicating a saturation effect. This suggests that increasing nitric acid concentration significantly promotes Fe(II) oxidation, but excessively high concentrations do not further accelerate the reaction, possibly due to reaching a reaction rate limit (Figure 3A). With the addition of acetic and citric acid (red, orange, yellow, green, and blue lines), the consumption rate of nitric acid is faster, indicating that organic acids promote nitric acid reduction. Without added organic acids (purple and pink lines), the consumption rate of nitric acid was slower. Notably, Fe(II) + nitrite (purple line) and Fe(II) + nitrite + nitrite (yellow line) showed a similar increase in nitric acid at later stages. However, the extent of nitrate reduction in Fe(II) + nitrite + nitrite (yellow line) was very low throughout the reaction (Figure 3B). The iron added in this experiment was in the form of ferrous citrate. Citrate ions dissociate in aqueous solution, increasing the citric acid concentration. We speculate that when the citric acid concentration in the medium reaches a lower level, it acts as an electron donor to oxidize Fe(II), leading to an increase in Fe(III) concentration. This is consistent with the result of Figure 2B. High concentrations of citric acid may inhibit nitric acid reduction. The control group with inactive cells (black line) indicates that cell activity is crucial for nitrate reduction.

These results indicate that nitric acid is highly effective as an electron acceptor in Fe(II) oxidation, particularly at higher concentrations. Meanwhile, organic acids as carbon sources significantly accelerate nitric acid reduction. These findings provide important insights into the role of microorganisms in nitrate reduction and have significant implications for the study of environmental biogeochemical processes.

**Figure 4:**
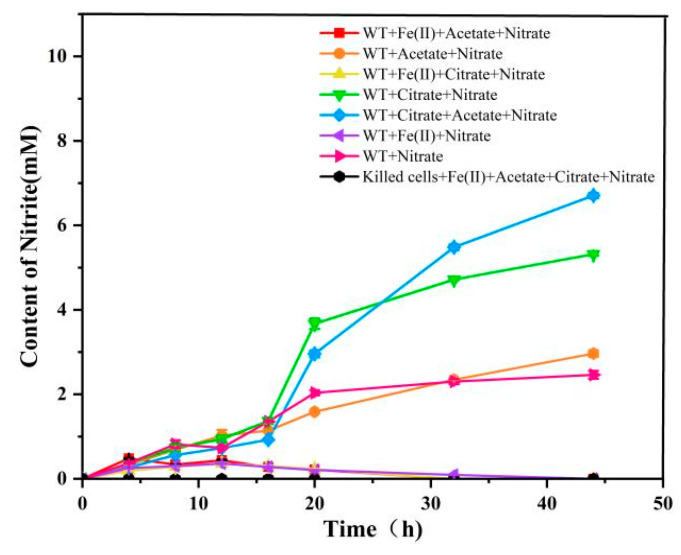
Dynamic changes in nitrite during nitrate reduction under different conditions. The curves shown in Figure 4 correspond to the nitrite production under the same conditions.

Nitrite accumulation in the WT + citrate + acetate + nitrate group (blue line) and the WT + citrate + nitrate group (green line) is significantly higher than in other groups, suggesting that in the absence of Fe(II), a lower proportion of nitrite is further reduced to secondary products in these groups. This may be due to the spontaneous oxidation of Fe(II) by nitrite. However, the electron donor used in the experiment is ferrous citrate, which was prepared by dissolving ferrous carbonate in citric acid. Adding it to the culture medium inevitably introduces interference from citric acid, and the results of the green and blue curves show that citric acid can act as an electron donor alone to provide electrons for reducing nitric acid, thereby producing nitrous acid. In this process, the electron acceptor is still nitric acid, but citric acid is preferentially utilized by bacteria. Therefore, there may be a competition between citric acid and Fe(II) as electron donors during the experimental process. The WT + acetate + nitrate group (orange line) and the WT + nitrate group (blue line) exhibit trends similar to the WT + citrate + acetate + nitrate and WT + citrate + nitrate groups, but with significantly lower nitrite accumulation. This could be due to the absence of citrate, supporting the hypothesis that citrate acts as an electron donor, providing electrons for further reactions with nitrate. The curves for WT + Fe(II) + acetate + nitrate (red line), WT + Fe(II) + citrate + nitrate (yellow line), and WT + Fe(II) + nitrate (purple line) indicate that in the presence of Fe(II), nitrite accumulation peaks are very low. This suggests that in the presence of Fe(II), nitrite is immediately reduced to other nitrogenous secondary compounds upon its formation. In all active bacterial groups, the nitrite accumulation rate decreases in the later stages of the experiment, further suggesting that nitrite is either reduced further or involved in other metabolic processes. The killed cells + Fe(II) + acetate + citrate + nitrate group (black line) serves as a control and shows almost no nitrite production, demonstrating that biological activity is essential (Figure 4). Overall, the presence of organic acids and Fe(II) significantly influences nitrite production and consumption. Organic acids likely act as electron donors, facilitating nitrate reduction to nitrite, while the addition of Fe(II) further accelerates this process. These dynamic changes are crucial for understanding nitrate reduction pathways and their environmental impact, particularly in relation to environmental engineering applications for microbial remediation of nitrate pollution.

#### 3.1.3. MR-1 Organic Acid Variation Curve

**Figure 5:**
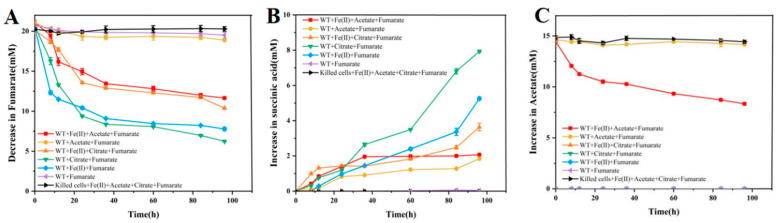
The variation curves of acetic acid, fumaric acid, and succinic acid during the reaction of Shewanella oneidensis MR-1 under different conditions. Acetic acid change curve (**A**), fumaric acid change curve (**B**), succinic acid change curve (**C**).

The results show fumarate consumption under various conditions during Fe(II) oxidation. The WT + citrate + fumarate group (green line) and the WT + Fe(II) + fumarate group (blue line) exhibit the highest fumarate consumption rates, likely due to Fe(II) and citrate acting as electron donors to enhance fumarate utilization. The WT + Fe(II) + citrate + fumarate group (orange line) and the WT + Fe(II) + acetate + fumarate group (red line) also show significant fumarate reduction, but at lower levels compared to the groups without added acetate (blue and green lines). Under normal conditions, acetate as a carbon source is expected to promote anaerobic respiration; however, this anomaly warrants further investigation. The WT + fumarate group (purple line) and the WT + acetate + fumarate group (yellow line) show almost no fumarate consumption, likely due to the absence of a complete electron transport chain, which prevents effective electron donor utilization (Figure 5A). In the results of succinic acid generation, corresponding to the groups with the highest fumarate consumption in Figure 5A, WT + citrate + fumarate (green line) and WT + Fe(II) + fumarate (blue line) exhibit the highest succinate production. Additionally, WT + Fe(II) + citrate + fumarate (orange line) and WT + Fe(II) + acetate + fumarate (red line) show an initial rapid increase in succinate, followed by a slower rise, likely related to iron equilibrium dynamics. The WT + acetate + fumarate group (yellow line) also displays minor succinate production, which may result from limited internal electron acceptors reducing a portion of fumarate in the presence of acetate. In contrast, the acetate + fumarate group (purple line) shows no significant oxidative respiration, potentially leading to bacterial lysis, which releases intracellular contents, including succinate, resulting in a detectable increase despite no fumarate consumption (Figure 5B). The consumption of acetic acid varies under different reaction conditions. In the WT + Fe(II) + acetate + fumarate group (red line), the complete electron transport chain enables normal bacterial metabolism, leading to acetate consumption. In contrast, the WT + acetate + fumarate group (yellow line) and the killed cells + Fe(II) + acetate + citrate + fumarate group (black line) show no significant acetate consumption, indicating a lack of bioactivity due to the absence of electron donors and acceptors (Figure 5C).

These results suggest that in environments rich in organic acids and Fe(II), fumarate and acetate metabolisms are highly active, leading to substantial succinate production [58]. The dynamic changes in these organic acids reflect complex microbial metabolic pathways, in which microorganisms utilize organic acids and Fe(II) to support growth and energy production. These findings have significant implications for understanding the role of microorganisms in environmental processes and biogeochemical cycles.

### 3.2. Mutant Strain Testing Section

#### 3.2.1. Ferrous Oxidation Curve of Mutant Strain

**Figure 6:**
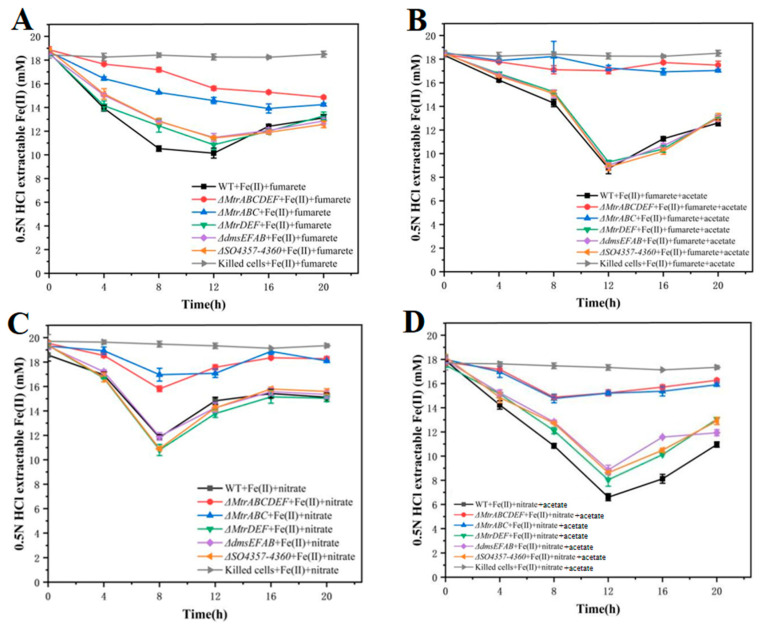
The oxidation curves of ferrous ions with fumaric acid (**A**,**B**) and nitric acid (**C**,**D**) as electron acceptors for *Shewanella oneidensis* MR-1 wild-type and various mutant strains. Two types of electron acceptors distinguish between acetic acid with (**B**,**D**) carbon-free sources. The gene clusters knocked out by each mutant strain are *MtrABC*, *MtrDEF*, *MtrABCDEF*, *dmsEFAB*, and *so4357–4360*.

Under conditions where fumarate serves as the electron acceptor, Δ*MtrDEF* (green), Δ*dmsEFAB* (yellow), Δ*so4357–4360* (purple), and WT (black) exhibit similar Fe(II) oxidation rates. However, Fe(II) oxidation is significantly reduced in Δ*MtrABCDEF* (red) and Δ*MtrABC* (blue) compared to the wild type, indicating that the *MtrABC* gene cluster is crucial for Fe(II) oxidation. This effect is more pronounced with the addition of acetate, a carbon source that enhances bacterial activity (Figure 6A,B).

Under conditions where nitrate serves as the electron acceptor, the mutant strains exhibit trends similar to those in Figure 6A,B regarding Fe(II) oxidation compared to the wild type. Notably, the Fe(II) oxidation rate in Δ*MtrABCDEF* and Δ*MtrABC* mutants is lower when nitrate is the electron acceptor compared to fumarate, further emphasizing the role of the *MtrABC* gene cluster in Fe(II) oxidation coupled with nitrate reduction. Additionally, the addition of acetate consistently enhances Fe(II) oxidation across all mutant strains, a trend observed in each mutant. The control group, killed cells (gray), shows almost no Fe(II) oxidation activity, confirming that biological activity is essential for Fe(II) oxidation (Figure 6C,D).

In summary, these results emphasize the crucial role of the *MtrABC* gene cluster in Fe(II) oxidation by *Shewanella oneidensis* MR-1. Moreover, the addition of acetate partially mitigates the negative impact of gene knockouts on Fe(II) oxidation, providing valuable insights for further exploration of metabolic pathways in these mutants.

#### 3.2.2. Variation Curves of Nitrate and Nitrite in Mutant Strains During the Reaction Process

**Figure 7:**
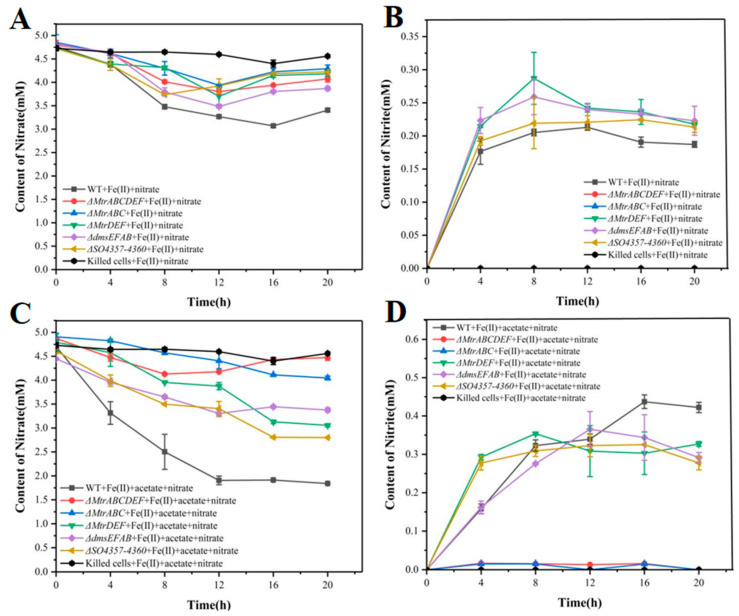
Changes in nitrate and nitrite content during nitrate reduction in wild-type and different mutant strains of *Shewanella oneidensis* MR-1. The gene clusters knocked out by each mutant strain are *MtrABC*, *MtrDEF*, *MtrABCDEF*, *dmsEFAB*, and *so4357–4360*. The nitrate reduction curves of each strain without carbon source (**A**), the nitrite generation curves of each strain without carbon source (**B**), the nitrate reduction curves of each strain with acetic acid as carbon source (**C**), and the nitrite generation curves of each strain with acetic acid as carbon source (**D**).

The result shows nitrate consumption and nitrite production during Fe(II) oxidation for wild-type and mutant strains without acetate as a carbon source. Compared to the wild type (gray), Δ*MtrABCDEF* (red) and Δ*MtrABC* (blue) exhibit significantly reduced nitrate reduction following the deletion of the *MtrABC* gene cluster, while the other mutants retain varying levels of nitrate reduction. This suggests that although *MtrDEF*, *DmsEFAB*, and *so4357–4360* contribute to nitrate reduction, their roles are less critical than those of *MtrABC*. Furthermore, in Δ*MtrABCDEF* (red) and Δ*MtrABC* (blue), almost no nitrite accumulates, likely due to the loss of sustained Fe(II) oxidation, which is necessary to drive nitrate reduction (Figure 7A,B). Consequently, any nitrate converted to nitrite undergoes further reduction or consumption, preventing nitrite accumulation [59].

With the addition of acetate, nitrate reduction is enhanced in all strains, including the wild type, except for Δ*MtrABCDEF* (red) and Δ*MtrABC* (blue). Nitrite accumulation also increases significantly across strains, likely due to acetate enhancing bacterial metabolic activity. These data indicate that different gene knockouts exhibit distinct efficiencies and dynamics in nitrate and nitrite metabolism, highlighting the roles of these genes in the nitrate reduction pathway (Figure 7C,D). The *MtrABC* cluster, in particular, directly impacts specific steps in the nitrate reduction pathway. These findings provide significant insights into the molecular mechanisms underlying nitrate reduction.

## 4. Discussion

### 4.1. Result Analysis

This study demonstrates the ability of *Shewanella oneidensis* MR-1 to perform nitrate reduction and Fe(II) oxidation (NRFO) under various conditions [60]. By comparing Fe(II) oxidation, nitrate reduction, and organic acid metabolism across different gene knockout mutants and wild-type strains, it was observed that both wild-type and mutant strains tested in this study exhibit Fe(II) oxidation capabilities in nitrate- or fumarate-containing environments. In the presence of organic acids such as acetate and fumarate, relatively high levels of iron oxidation activity were observed, suggesting that organic acids play a significant role in promoting iron oxidation [61]. The production and accumulation of acetate and succinate indicate that *Shewanella oneidensis* derives energy from organic acid metabolism while simultaneously oxidizing iron [62]. Fumarate consumption reflects an essential aspect of cellular metabolism, acting as an electron acceptor to support cellular energy demands, particularly during iron reduction processes. These results indicate that nitric acid is highly effective as an electron acceptor in Fe(II) oxidation, particularly at higher concentrations. Meanwhile, organic acids as carbon sources significantly accelerate nitric acid reduction. These findings provide important insights into the role of microorganisms in nitrate reduction and have significant implications for the study of environmental biogeochemical processes.

In addition, the *MtrABC* gene cluster knockout strains exhibited substantially reduced iron oxidation compared to the wild type, emphasizing the crucial role of these gene clusters within the electron transport chain. Although nitrate reduction efficiency varied among mutants, all strains tested in this study reduced nitrate, indicating a relatively stable nitrate reduction pathway in *Shewanella oneidensis*. However, some differences in nitrite processing were observed among the mutants during nitrate reduction, which may result from alterations in nitrate reductase activity or metabolic pathway adjustments [63]. These results emphasize the crucial role of the *MtrABC* gene cluster in Fe(II) oxidation by *Shewanella oneidensis* MR-1. Moreover, the addition of acetate partially mitigates the negative impact of gene knockouts on Fe(II) oxidation, providing valuable insights for further exploration of metabolic pathways in these mutants.

These observations suggest that *Shewanella oneidensis* MR-1 exhibits significant metabolic flexibility under different environmental pressures, allowing it to adjust its metabolic pathways to adapt to various conditions. Although knockouts of the *MtrDEF*, *dmsEFAB*, and *so4357–4360* gene clusters affected specific metabolic pathways, the bacteria maintained essential functions, such as electron transport and energy generation, through compensatory pathways. Similarly, whether the metabolic compensation mechanism of the genes still exists after the addition of other substances needs to be further investigated. The *MtrABC* cluster, however, appears irreplaceable, highlighting its unique role. This compensatory mechanism may be crucial for understanding and utilizing these microorganisms in environmental biotechnology, particularly in bioremediation and bioenergy production.

### 4.2. Mechanism Exploration

**Figure 8:**
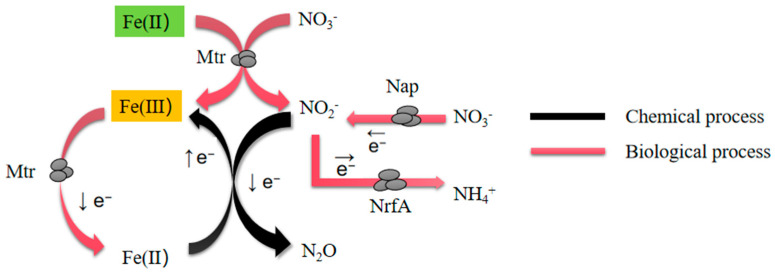
Chemical and biological processes of iron (Fe) and nitrogen oxides (NOx) in environmental microbial processes. The arrows in the figure represent the direction of electron flow, which is consistent with the direction of biological and chemical processes.

Nitrate reduction and ferrous oxidation (NRFO) is a complex microbial process involving multiple enzyme systems and metabolic pathways. We have mapped the mechanism of the interaction between iron and nitrogen redox processes using both biological and chemical approaches (Figure 8). The overall process is driven by microbial and chemical activities. Microorganisms oxidize Fe(II) to Fe(III), releasing electrons (e^−^) in the process. These electrons are not lost; instead, they participate in further biochemical reactions [64]. The released electrons are subsequently transferred to nitrate (NO_3_^−^) via microbial enzymes (labeled as Mtr in the figure), reducing it to nitrite (NO_2_^−^). This reduction is microbially mediated, represented by the red arrow labeled NrfA, and is crucial in anaerobic environments as it regulates the redox state of iron and influences key steps in the nitrogen cycle [65].

This diagram illustrates the interlinked chemical and biological processes involved in the cycling of iron and nitrogen oxides (NO_x_) in environmental microbial systems. The iron cycle involves the interconversion of ferrous iron (Fe(II)) and ferric iron (Fe(III)). Fe(II) is oxidized to Fe(III) through the release of electrons mediated by Mtr, a membrane-associated electron transport protein, in a biologically driven process (denoted by red arrows). Conversely, Fe(III) can be chemically reduced back to Fe(II) via abiotic reactions, completing the iron redox cycle (black arrows). Once generated, nitrite (NO_2_^−^) has two potential fates. First, nitrite can be reduced to ammonia (NH_4_^+^) via the microbial enzyme NrfA, a common nitrogen assimilation pathway essential for maintaining nitrogen balance within ecosystems [66]. Alternatively, NO_2_^−^ can undergo abiotic reduction to nitrous oxide (N_2_O), a potent greenhouse gas. This process typically occurs under low-oxygen conditions and is often coupled with iron reduction, illustrating the complex interplay between iron and nitrogen cycles. The nitrogen cycle depicted focuses on the reduction of nitrate and nitrite through microbial enzymatic activity. Nitrate is reduced to nitrite via Nap (nitrate reductase), releasing electrons in the process. Nitrite can then be further reduced to ammonium through NrfA (cytochrome nitrite reductase), or to intermediates such as nitric oxide and nitrous oxide during denitrification processes. This integration of nitrogen redox transformations with iron cycling demonstrates the intricate connections between microbial metabolisms and geochemical pathways in natural environments. Biological processes are represented with red arrows, while chemical processes are indicated with black arrows. In *Shewanella oneidensis* MR-1, this process is noteworthy because it involves simultaneous Fe(II) oxidation and nitrate reduction, using nitrate as an electron acceptor [67]. The results of this study reveal that gene regulation is crucial for modulating NRFO. Specific genes, such as those in the *MtrABC* cluster, likely affect the efficiency of both iron oxidation and nitrate reduction. These genes encode proteins that transfer electrons from within the cell to the exterior, linked to outer membrane cytochromes, and are crucial for electron transfer in both Fe(II) oxidation and nitrate reduction. Knockouts of these gene clusters reduce electron transfer efficiency, thereby affecting NRFO rates [2]. Observations of nitrite and organic acids (e.g., succinate and acetate) suggest that these intermediates play key roles in regulating electron flow and maintaining cellular energy balance. Nitrite may temporarily accumulate as an intermediate electron acceptor during nitrate reduction, while organic acids may provide additional electron donors or contribute to electron transport chain regulation [68].

Environmental factors such as pH, temperature, and the type and concentration of organic matter can impact NRFO efficiency. For example, organic acids may act as both electron donors and pH modifiers, indirectly influencing electron transfer and iron solubility [69]. Under specific conditions, such as limited organic matter or high nitrate concentrations, bacteria may adjust their metabolic pathways to optimize energy production and storage. This may explain why bacteria can maintain NRFO activity to some extent even under gene knockout conditions, through compensatory mechanisms [48]. Together, these factors define the NRFO performance of *Shewanella oneidensis* MR-1 under various environmental conditions, highlighting its potential for microbial-based environmental remediation and bioenergy production. Fe(II) oxidation impacts both the biogeochemical cycling of iron and the reduction of nitrogen oxides via electron transfer pathways. Investigating these processes is crucial for understanding microbial ecosystems in soil and water, pollutant migration, and greenhouse gas dynamics. The coupling of iron and nitrogen cycles in microbially mediated environmental reactions reveals complex biogeochemical processes and highlights areas for further exploration in future research [66].

## 5. Conclusions

This study employed microcosm cultures of *Shewanella*, a model nitrate-reducing and Fe(II)-oxidizing microorganism. Molecular biology techniques were then used to elucidate the relative contributions of *Shewanella*-mediated NRFO processes and chemical denitrification in Fe(II) oxidation. By examining the transformations of iron in specific forms (e.g., Fe(II), Fe(III)) and the accompanying nitrogen cycling under anaerobic conditions, this research advances our understanding of microbial contributions to iron cycling. The primary objective was to uncover the mechanisms of NRFO processes under anoxic conditions, with a focus on the distinct roles of microbial and chemical denitrification in Fe(II) oxidation. This study also aimed to reveal the interconversion of iron and nitrogen elements across different redox states. These findings significantly enhance our understanding of specific microbial functions, such as electron transport and denitrification, within environmental iron cycling.

In natural ecosystems, iron cycling plays a crucial role in maintaining ecological balance. This study demonstrates that NRFO is not merely a singular microbial process but involves a complex network of biological and chemical reactions. Microbes reduce nitrate to nitrite, releasing electrons that subsequently oxidize Fe(II) to Fe(III)—a process particularly important under anoxic conditions. Chemical denitrification provides a parallel pathway, involving various chemical reactions that further oxidize or reduce nitrogen compounds. By delineating these processes, this study highlights the unique contributions of microbes to iron redox cycling and demonstrates how iron and nitrogen are interconnected, driving biogeochemical cycles through both biological and chemical interactions. At the same time, the electron acceptors we studied focused only on nitrate and fumarate, and the electron donors were similarly studied only for iron, leaving our conclusions unknown as to whether other electron donors and electron acceptors have the same effect on the above mechanism.

The significance of this research extends to a broader understanding of global biogeochemical cycles. NRFO processes are not limited to controlled laboratory settings; they have broad implications for natural environments, influencing the cycling of elements such as carbon, nitrogen, and iron. For instance, various oxidation states of iron are widely distributed in soil and aquatic systems, where NRFO regulates these forms, affecting the solubility and bioavailability of iron. Also, the experiment did not fully consider all environmental factors. It is undeniable that there are differences between the natural environment and the laboratory environment. Therefore, the above experiments can be further improved and applied to natural conditions for further verification in the future. Additionally, nitrogen reduction is directly related to the generation and emission of greenhouse gases, such as nitrous oxide (N_2_O), a potent greenhouse gas with significant effects on climate change. Nitrous oxide (N_2_O) is a potent greenhouse gas with significant environmental implications due to its high global warming potential and role in stratospheric ozone depletion. Emissions of N_2_O originate from both natural and anthropogenic sources. Natural emissions occur primarily from microbial processes in soils and oceans, including nitrification and denitrification, while anthropogenic emissions arise from activities such as agricultural fertilization, fossil fuel combustion, and industrial processes. Excessive nitrogen fertilization in agriculture is the largest contributor, as it enhances microbial production of N_2_O in soils. Once released, N_2_O contributes to the greenhouse effect by trapping heat in the atmosphere, exacerbating global warming. Moreover, it participates in stratospheric reactions that deplete ozone, reducing the protective layer that shields Earth from harmful ultraviolet radiation. The environmental consequences of increased N_2_O emissions include rising global temperatures, more frequent extreme weather events, disruptions in ecosystems, and adverse impacts on biodiversity. An in-depth understanding of NRFO processes can enable more accurate predictions and management of elemental cycles in the environment, thereby contributing to climate change research and environmental protection [70,71,72].

Finally, this study offers new insights and applications for environmental remediation. Understanding how microbes facilitate iron oxidation and nitrogen reduction under anaerobic conditions can guide the development of effective pollution control strategies. For example, managing NRFO processes in eutrophic water bodies could help control nitrogen and iron levels, thereby reducing pollutant concentrations and improving water quality. In soil remediation, leveraging NRFO could stabilize iron forms, reduce heavy metal mobility, and consequently lower ecological toxicity. In water immersion, sewage environments rich in organic matter and nitrate, under nitrate conditions, the trivalent iron produced by the oxidation of ferrous iron will form rust in iron water pipes, and the formation of this rust will further erode elemental iron to form divalent iron, thereby forming a positive feedback and intensifying the damage to related iron products. Therefore, by combining our research results with bioremediation and interference methods, we can break this feedback and control nitrate pollution or iron cycling in both natural and artificial environments. This research thus holds substantial scientific value and offers critical theoretical support and practical guidance for environmental management and ecological restoration.

## Data Availability

Data is contained within the article or supplementary material.

## Supplementary Materials

The following supporting information can be downloaded at https://www.mdpi.com/article/10.3390/microorganisms12122454/s1: Figure S1. Total iron variation curves of *Shewanella oneidensis* MR-1 under different conditions using fumaric acid (A) and nitric acid (B) as electron acceptors; Figure S2. The content of the reaction starting point, midpoint, and endpoint of nitric acid metabolites such as nitrous oxide, ammonia nitrogen, and total nitrogen in the process of ferrous oxidation; Figure S3. Diagram of changes in the experimental culture medium device at different time points during the reaction process; Table S1: Different treatment groups of MR-1 wild-type strain under two electron acceptor conditions; Table S2: Different treatment groups of MR-1 mutant strains under two electron acceptor conditions; Table S3. Experimental instrument information; Table S4. Chemical reagent information.
