# Supplementary material for: Biological and Chemical Processes of Nitrate Reduction and Ferrous Oxidation Mediated by Shewanella oneidensis MR-1
Source: Microorganisms. 2024 Nov 29;12(12):2454. doi: 10.3390/microorganisms12122454 (PMC11676297; doi:10.3390/microorganisms12122454)
Supplement: Supplementary file 1 [file microorganisms-12-02454-s001.zip › microorganisms-3288601-supplementary.pdf]

Supplementary documents:

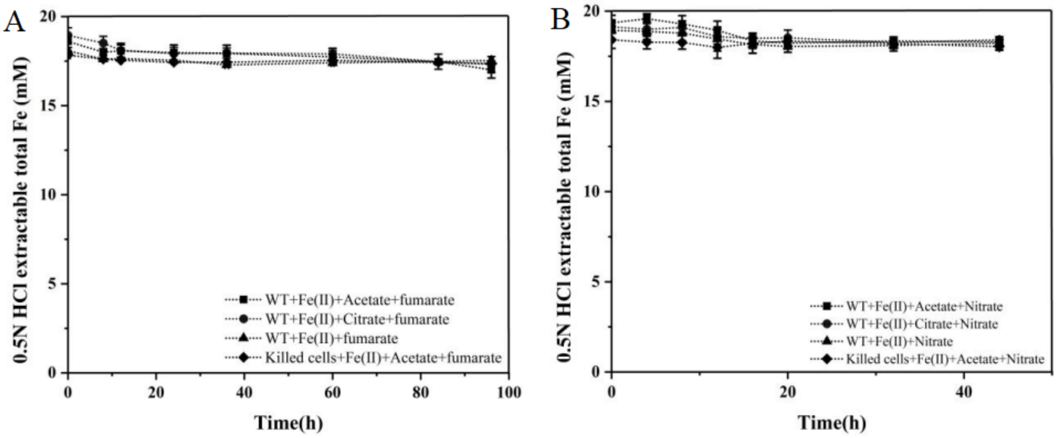

Figure S1. Total iron variation curves of *Shewanella oneidensis* MR-1 under different conditions using fumaric acid (A) and nitric acid (B) as electron acceptors

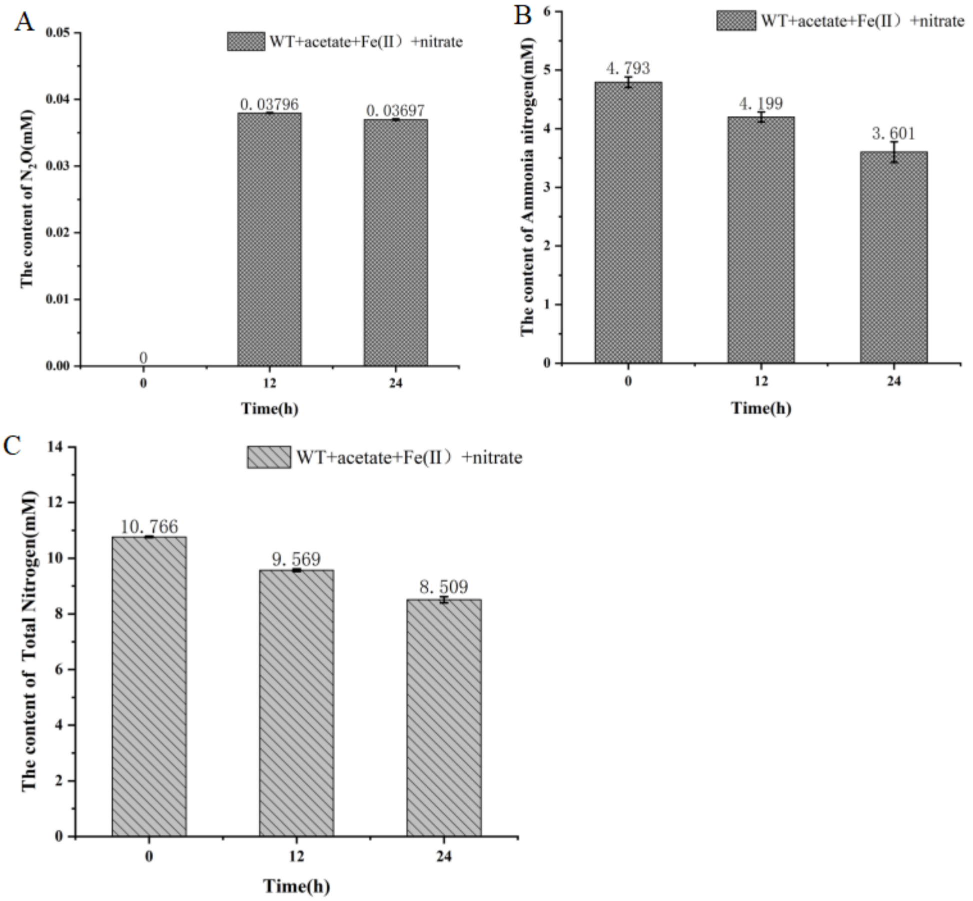

Figure S2. The content of reaction starting point, midpoint, and endpoint of nitric acid metabolites such as nitrous oxide (A), ammonia nitrogen (B), and total nitrogen (C) in the process of ferrous oxidation

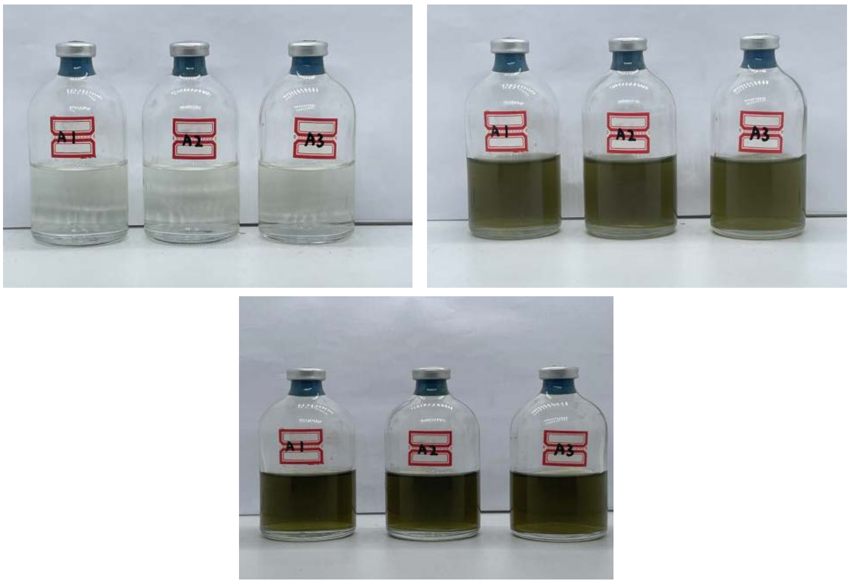

Figure S3. Diagram of changes in experimental culture medium device at different time points during the reaction process

Table S1. Different treatment groups of MR-1 wild-type under two electron acceptor con-ditions.

Electron Acceptor	Strain	Medium Components
Fumarate	*Shewanella oneidensis* MR-1 WT	Cell+Fe(II)+Acetate+Fumarate
		Cell+Acetate+Fumarate
		Cell+Fe(II)+Citrate+Fumarate
		Cell+Citrate+Fumarate
		Cell+Fe(II)+Fumarate
		Cell+Fumarate
	Killed cell	Cell+Fe(II)+Citrate+Fumarate
		Cell+Fe(II)+Acetate+Fumarate
Nitrate	*Shewanella oneidensis* MR-1 WT	Cell+Fe(II)+Acetate+Nitrate
		Cell+Acetate+Nitrate
		Cell+Fe(II)+Citrate+Nitrate
		Cell+Citrate+Nitrate
		Cell+Fe(II)+Nitrate
		Cell+Nitrate
	Killed cell	Cell+Fe(II)+Citrate+Nitrate
		Cell+Fe(II)+Acetate+Nitrate

Table S2. Different treatment groups of MR-1 mutant strains under two electron acceptor conditions.

Electron Acceptor	Strain	Medium Components
Fumarate	WT	Cell+Fe(II)+fumarete
		Cell+Fe(II)+Acetate+fumarate
	Δ*MtrABCDEF*	Cell+Fe(II)+fumarete
		Cell+Fe(II)+Acetate+fumarate
	Δ*MtrABC*	Cell+Fe(II)+fumarete
		Cell+Fe(II)+Acetate+fumarate
	Δ*MtrDEF*	Cell+Fe(II)+fumarete
		Cell+Fe(II)+Acetate+fumarate
	Δ*dmsEFAB*	Cell+Fe(II)+fumarete
		Cell+Fe(II)+Acetate+fumarate
	Δ*so4357-4360*	Cell+Fe(II)+fumarete
		Cell+Fe(II)+Acetate+fumarate
Nitrate	WT	Cell+Fe(II)+Nitrate
		Cell+Fe(II)+Acetate+Nitrate
	Δ*MtrABCDEF*	Cell+Fe(II)+Nitrate
		Cell+Fe(II)+Acetate+Nitrate
	Δ*MtrABC*	Cell+Fe(II)+Nitrate
		Cell+Fe(II)+Acetate+Nitrate
	Δ*MtrDEF*	Cell+Fe(II)+Nitrate
		Cell+Fe(II)+Acetate+Nitrate
	Δ*dmsEFAB*	Cell+Fe(II)+Nitrate
		Cell+Fe(II)+Acetate+Nitrate
	Δ*so4357-4360*	Cell+Fe(II)+Nitrate
		Cell+Fe(II)+Acetate+Nitrate

Table S3. Experimental instrument information

Instrument Name	Brand
Ultra-low Temperature Freezer	Thermo Fisher Scientific
Medical Refrigerator	Haier
Analytical Balance	Mettler Toledo
Refrigerated Centrifuge	Eppendorf
Mini Centrifuge	Baygene
Vortex Mixer	QiLinBeiEr
Autoclave	Shanghai Shenan
Biosafety Cabinet	Thermo Fisher Scientific
Clean Bench	Thermo Fisher Scientific
PCR Machine	Bio-Rad

pH Meter	Mettler Toledo
Ultrapure Water System	Milli-Q
Electrophoresis Apparatus	Bio-Rad
Gel Imaging System	Thermo Fisher Scientific
All-temperature Oscillator Incubator	Tiancheng
Magnetic Stirrer	Mettler Toledo
Anaerobic Glove Box	Coylab
UV Spectrophotometer	Thermo Fisher Scientific
Handheld Centrifuge	Dragonlab
Oven	Shanghai Jinghong
Deoxygenation Device	Self-assembled
Microwave	Midea
HPLC Liquid Chromatograph	Shimadzu
IC Ion Chromatograph	Shimadzu

Table S4. Chemical reagent information

Reagent Name	Grade	Manufacturer
Hydrochloric Acid (HCl)	Superior Grade	Sinopharm Chemical Reagent Co., Ltd.
Sodium Nitrate (NaNO3)	Analytical Grade	Shanghai Shenggong Co.
Sodium Nitrite (NaNO2)	Analytical Grade	Shanghai Shenggong Co.
Trisodium Citrate	Analytical Grade	Shanghai Shenggong Co.
Sodium Sulfate (NaSO4)	Analytical Grade	Shanghai Shenggong Co.
Potassium Chloride (KCl)	Analytical Grade	Shanghai Shenggong Co.
Sodium Bicarbonate (NaHCO3)	Analytical Grade	Shanghai Shenggong Co.
Ammonium Chloride (NH4Cl)	Analytical Grade	Shanghai Shenggong Co.
Anhydrous Monosodium Phosphate (NaH2PO4)	Analytical Grade	Shanghai Shenggong Co.
EDTA Electrophoresis Buffer	Analytical Grade	Shanghai Shenggong Co.
Sodium Hydroxide (NaOH)	Superior Grade	Sinopharm Chemical Reagent Co., Ltd.
Ferric Chloride (FeCl3)	Analytical Grade	Sinopharm Chemical Reagent Co., Ltd.
2×ES Taq PCR Mastermix	Superior Grade	Kangwei Century
Phenol	Organic Pure	Takara
2000 DNA Maker	Superior Grade	Takara
5000 DNA Maker	Superior Grade	Takara
10000 DNA Maker	Superior Grade	Takara

1kb DNA Maker	Superior Grade	Takara
